# Association of social support with cognition among older adults in China: A cross-sectional study

**DOI:** 10.3389/fpubh.2022.947225

**Published:** 2022-09-26

**Authors:** Benchao Li, Yan Guo, Yan Deng, Siqi Zhao, Changfeng Li, Jiajia Yang, Qiuying Li, Yaqiong Yan, Fang Li, Xiaonuan Li, Shuang Rong

**Affiliations:** ^1^Department of Nutrition Hygiene and Toxicology, School of Public Health, Medical College, Academy of Nutrition and Health, Wuhan University of Science and Technology, Wuhan, China; ^2^Wuhan Centers for Disease Prevention and Control, Wuhan, China; ^3^Wuhan Municipal Health Commission, Wuhan, China

**Keywords:** cognitive function, cognitive impairment, older adults, social support, cross-sectional study

## Abstract

**Objective:**

This study aimed to examine the relationship between social support and its sub-domains and cognitive performance, and the association with cognitive impairment among older adults in China.

**Design:**

A cross-sectional study.

**Setting and participants:**

We included 865 community-based individuals aged 65 and above from Hubei province, China.

**Methods:**

The level of social support was evaluated using the social support rating scale (SSRC). The Mini-Mental State Examination was adopted to assess cognitive function, and its cut-offs were used to determine cognitive impairment among the participants. Multiple linear regression models and logistic regression models were used to estimate the β and odds ratios (*ORs*) and their 95% CIs, respectively.

**Results:**

The participants were divided into quartiles 1–4 (Q1–Q4), according to the total scores of SSRC. After adjusting for sociodemographic characteristics, lifestyle factors, and history of diseases, for MMSE scores, compared to these in Q1, the β of Q2–Q4 were −0.22 (−0.88, 0.43), 0.29 (−0.35, 0.94), and 0.86 (0.19, 1.53), respectively; For cognitive impairment, the *ORs* of Q2–Q4 were 1.21 (0.80, 1.82), 0.62 (0.40, 0.94), and 0.50 (0.32, 0.80), respectively. Considering SSRC scores as the continuous variable, per 1-unit increase, the β was 0.05 (0.02, 0.09) for the cognitive score, and the *OR* was 0.95 (0.92, 0.98) for cognitive impairment. In addition, higher levels of both subjective support and support utilization were related to better MMSE performance and lower risks of cognitive impairment.

**Conclusion and implications:**

Among the older adults in China, as expected, there is a positive relationship between social support and cognitive performance, and high levels of social support, particularly in support utilization, were related to low risks of cognitive impairment. More social support should be provided in this population to improve cognitive function and reduce the risks of cognitive impairment.

## Introduction

With the rapid aging of the population over the world, the diseases of cognitive impairment, including mild cognitive impairment (MCI) and dementia, are causing a tremendous burden on the economy and health (1, 2). MCI is a disorder characterized by impairment of memory, learning difficulties, and reduced ability to concentrate on a task for more than brief periods, and has a high risk of progressing to dementia (3). It is estimated that there are 15.07 million people with dementia and 38.77 million people with MCI in China (4). Given no effective treatment for dementia, the efforts on modifiable influencing factors are important for improving cognitive function and preventing diseases of cognitive impairment among older adults.

For an individual, social support is the composition of material and spiritual support from various people and organizations, including family members, friends, neighbors, colleagues, and governmental and non-governmental organizations. Given that the resources to keep alive, such as food, daily supplies, and emotional support, largely depend on the family members and other for older adults, social support may play a crucial role in the health outcomes of older adults. In addition, a previous study showed that common comorbidities, such as hypertension, diabetes, glycemic variability, and dyslipidemia were linked to cognitive decline (5–7). Social support in the older adult may be beneficial to controlling blood pressure and lipids well and keeping blood glucose stable (8–11). Several studies have explored the associations of emotional and instrumental support with cognitive function and brain image among adults in the US (12–15), UK (16), Dutch (17), Mexican (18), and South Africa (19). The results from these studies (12–23) were conflicting due to the heterogeneities in study design, sample size, and study population. In addition, social support was often divided into different aspects (24), such as objective support from living materials, subjective support from emotional networks, and support utilization indicating the ability to get others' support. However, there were limited studies investigating the relationship between social support and cognition in China.

In this study, by a specialized questionnaire for assessing different aspects of social support, we conducted a cross-sectional study and examined the association of social support and its sub-domains with cognition among older adults aged 65 and above in China.

## Methods

### Study population and data collection

**Figure 1:**
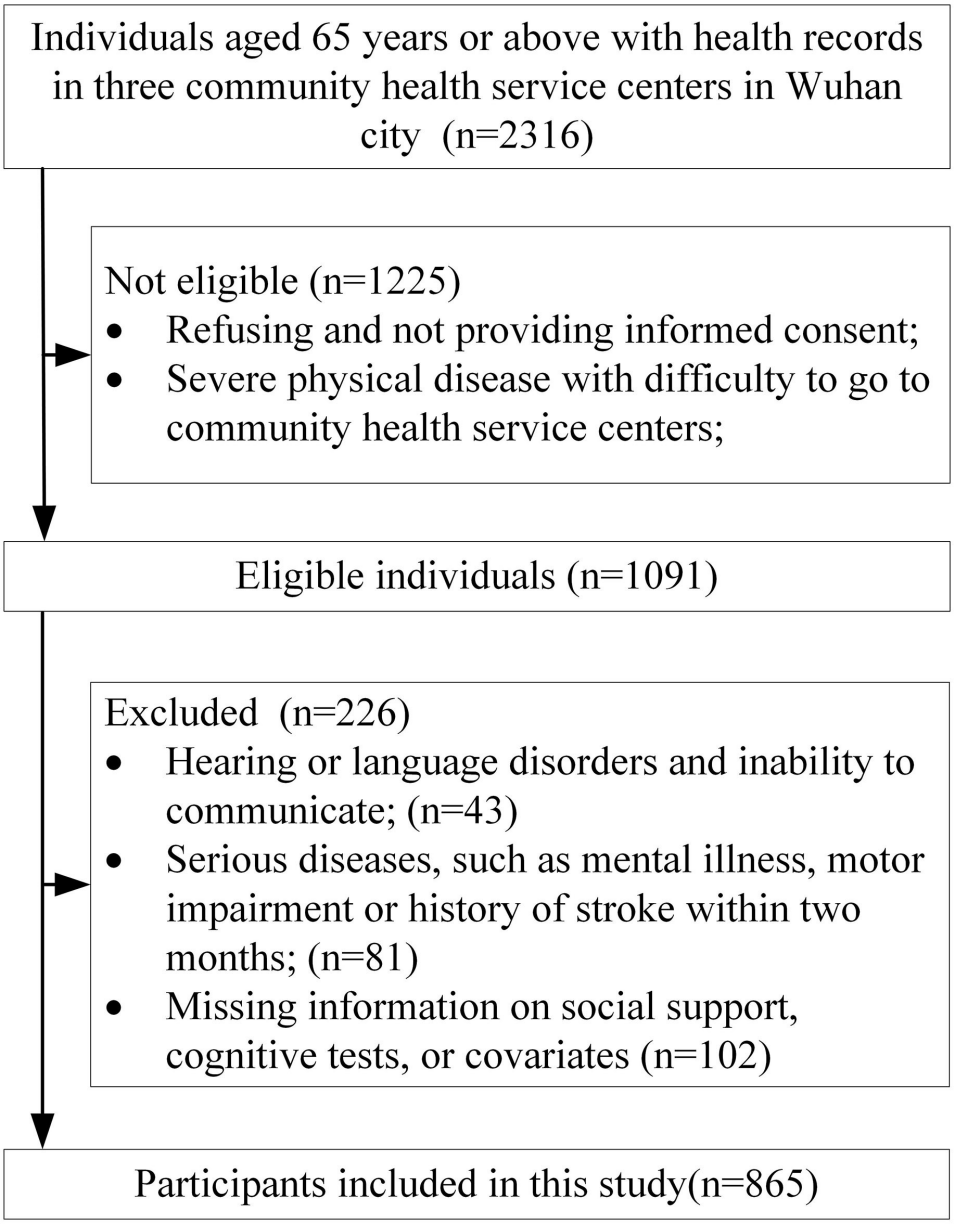
The flow chart of the inclusion and exclusion of study population.

Participants were recruited from Wuhan city, China. Eligible participants aged 65 and above volunteered to participate in this study and could provide informed consent and did not have severe physical diseases and were able to go to community health service centers. The following exclusion criteria were applied: (1) Hearing or language disorders and inability to communicate. (2) Severe diseases, such as mental illness, motor impairment, or history of stroke within two months. (3) Missing information on social support, cognitive test, or other covariates. We used following method to calculate the sample size, *n* = *Z*_*a*/2_^2^^*^ *P* ^*^ *(1-P) /* δ ^2^. According to previous studies (25, 26) on cognitive function among older adults in Hubei province, China, the prevalence of cognitive impairment was set as 25% in this study. In addition, Z _a/2_ was set as 1.96, δ (allowable error) was set as 3%, and the required sample size was calculated to be 800. Cluster sampling methods were used for the survey. We first performed random sampling methods to select three community health service centers in Wuhan city, the residents whose health records were registered in health service centers were recruited. With the inclusion and exclusion criteria, a total of 865 older adults aged 65 and above were used as the final analytic sample. The detailed flowchart was presented in Figure 1.

Baseline data on socio-demographic and background variables (including general demographic characteristics, lifestyle factors, and history of diseases) were collected using a self-made questionnaire. The interviewers underwent extensive training as well as periodic certification. One-to-one interview in a quiet room was employed to complete all questionnaires. This study was approved by the Medical Ethics Committee of Medical College, Wuhan University of Science and Technology (No. WUSTMC-201942). Written informed consent was obtained from all participants.

### Assessment of cognitive function

Mini-Mental State Examination (MMSE) originated from Folstein et al. in 1975 (27), and has 13 items, including orientation, attention, language, immediate recall, delayed recall, and construction, and the scores range from 0 to 30 points. High scores indicate good cognitive performance. The Chinese version of MMSE was employed to assess the cognitive function of participants in this study. The previous study has shown good reliability (28) and validity (29) of the Chinese version of the Mini-Mental State Examination. According to a previous validation study on the Chinese version of MMSE (29), the cut-off points of dementia and MCI were set as 16/17 and 19/20 for illiteracy, 19/20 and 24/25 for primary school, and 23/24 and 27/28 for junior high school and above in this population. The participants screened with dementia and MCI were considered to be with cognitive impairment.

### Measurement of social support score

The score of social support was measured by the social support rating scale (SSRS) (24). As shown in Table 1, the SSRS consists of three sub-domains such as objective support, subjective support, and support utilization, its total scores range from 12 to 66, and higher scores indicate better social support. Cronbach's a coefficient, split-half correlation coefficient, and test-retest correlation coefficient of SSRS were 0.821, 0.875, and 0.829, respectively, indicating high reliability (30). A previous study also showed high-construct validity and content validity (31). As a questionnaire to evaluate social support, SSRC has been widely accepted and used in epidemiological studies across China (32).

**Table 1:** The questions, options, and points of social support rating scale (SSRC)^a^.

**Questions**	**Options**	**Single/multiple, points**
1. What number of friends who could provide the support you have now?	1. Nobody 2. 1–2 people 3. 3–5 people 4. 6 people or above	Single, 1–4 points
2. In the past year, you are	1. Living far from family member, and living along 2. No fixed shelter and living with strangers frequently 3. Living with friends, colleagues, and classmates 4. Living with family numbers	Single, 1–4 points
3. The relationship between you and neighborhoods	1. Never care 2. A little of concern 3. Obvious concern from some neighborhoods 4. Lots of concern from many neighborhoods	Single, 1–4 points
4. The relationship between you and colleagues	1. Never care 2. A little of concern 3. Obvious concern from some colleagues 4. Lots of concern from many colleagues	Single, 1–4 points
5.1 The support and care from		
(1) Spouse	1. No 2. Rare 3. General 5. Complete	Single, 1–4 points
(2) Parents	1. No 2. Rare 3. General 6. Complete	Single, 1–4 points
(3) Kids	1. No 2. Rare 3. General 7. Complete	Single, 1–4 points
(4) Brothers and sister	1. No 2. Rare 3. General 8. Complete	Single, 1–4 points
(5) Other family members, such as the brother's wife	1. No 2. Rare 3. General 4. Complete	Single, 1–4 points
6. The resources of financial support and solving questions when you are in trouble	1. No any source 2. Spouse 3. Other family members 4. Friends 5. Relatives 6. Colleagues 7. Organizations in work 8. Organizations from parties and other governmental organizations 9. Religious and social organizations 10. Others	Multiple, 0–9 points
7. The resources of care and comfort when you are in trouble	1. No source 2. Spouse 3. Other family members 4. Friends 5. Relatives 6. Colleagues 7. Organizations in work 8. Organizations from parties and other governmental organizations 9. Religious and social organizations 10. Others	Multiple, 0–9 points
8. The ways of pouring out when you are annoyed	1. Never pouring out to others 2. Pouring out to one or two close people 3. Pouring out when friends talk with me for initiative 4. Pouring out to others for the initiative to get support and understanding	Single, 1–4 points
9. The ways of asking for help when you are annoyed	1. Never asking for help from others 2. Rarely asking for help from others 3. Sometimes asking for help from others 4. Often asking for help from others	Single, 1–4 points
10. How often to participate in activities from all kinds of organizations, including working organizations, organizations from parties and other governmental organizations, religious and social organizations, and others	1. Never 2. Rarely 3. Sometimes 4. Often	Single, 1–4 points

a The SSRS was divided into three domains: objective support (questions 2, 6, and 7), subjective support (questions 1, 3, 4, and 5), and level of support utilization (questions 8, 9, and 10).

### Socio-demographic characteristics, lifestyle factors, and history of diseases

According to the previous studies (33, 34), the socio-demographic characteristics, lifestyle factors, and history of diseases were considered covariates. Socio-demographic characteristics included age, sex, education level, and marital status. Education levels were divided into three categories: 0–6 years, 7–9 years, and ≥10 years. Marital status was categorized as married and non-married (single, divorced, or widowed). Lifestyle factors included smoking status and alcohol intake, which were defined as yes or no based on self-reported information. History of disease included obesity, hypertension, and diabetes. Body mass index (BMI) was calculated using weight to divide the square of height, and participants with a BMI < 18.5 kg/m^2^ were considered underweight, between 18.5 and 23.9 kg/m^2^ were considered normal, between 24 and 27.9 kg/m^2^ were considered overweight, and ≥ 28 kg/m^2^ were considered obese (35). Hypertension was defined as at least one of followed conditions: self-reported history of diagnosed hypertension, having anti-hypertension drugs or measured systolic blood pressure ≥140 mm Hg, or diastolic blood pressure ≥90 mm Hg (36). Diabetes was defined as at least one of the following conditions: self-reported history of diagnosed diabetes, having glucose-lowering medications or fasting blood glucose level of ≥7 mmol/L (37).

### Statistical analysis

Differences between groups in demographic variables were examined using ANOVA analysis for continuous variables, and Pearson's chi-square test for categorical variables. Multiple linear regressions were performed to calculate the partial regression coefficient between the social support and MMSE scores. Unconditional logistic regression models were used to examine the association of social support with cognitive impairment by evaluating the odds ratio. Besides, the linear relationship and odds ratio between scores of three domains of social support were also examined. The covariates were controlled in model 1: adjusted for age and sex; model 2, further adjusted for education level, marriage, smoking, and drink; model 3, further adjusted for BMI categories, hypertension, and diabetes. All reported *P*–values were two-tail, and *P* < 0.05 was considered to be statistically significant. All statistical analyses were performed using SPSS 19.0 (SPSS Inc., Chicago, IL, USA).

## Results

### Characteristics of participants

The mean age in this study was 70.44 years old, and 53.18% were women. There were 264 participants with cognitive impairment, with a 30.52% of cognitive impairment prevalence. The demographic data and lifestyle factors were presented in Table 2 according to the participants with the quartiles 1–4 of social support scores. The participants with higher support scores were more likely to have lower age, higher MMSE scores, higher education levels, and be married. There was no significant difference in sex, smoking, drinking, BMI, hypertension, and diabetes among these groups.

**Table 2:** Baseline characteristics of study population according to quartiles of social support scores^a^.

	**Social support**					
**Characteristic**	**Quartile 1 (12–28)**	**Quartile 2 (29–32)**	**Quartile 3 (33–36)**	**Quartile 4 (37–53)**	***F/χ^2^***	***P*-value**
**Sample size (** ***n*** **)**	223	197	234	211		
**Support**, ***mean*** **(*****SD*****)**	25.2 (2.9)	30.5 (1.1)	34.5 (1.2)	40.8 (3.5)	1603.16	< 0.001
**MMSE**, ***mean*** **(*****SD*****)**	25.8 (4.2)	25.9 (4.3)	26.8 (3.8)	27.5 (2.7)	9.49	< 0.001
**Age**, ***mean*** **(*****SD*****)**	71.6 (5.2)	70.8 (5.2)	69.9 (4.3)	69.4 (4.6)	9.08	< 0.001
**Sex**, ***n*** **(%)**					3.11	0.375
Male	107 (50.0)	94 (47.7)	116 (49.6)	88 (41.7)		
Female	116 (52.0)	103 (52.3)	118 (50.4)	123 (58.3)		
**Educational level**, ***n*** **(%)**					24.36	< 0.001
0–6 years	114 (51.2)	85 (43.2)	82 (35.0)	66 (31.3)		
7–9 years	65 (29.2))	66 (33.5)	82 (35.0)	73 (34.6)		
≥10 years	44 (19.7)	46 (23.4)	70 (29.9)	72 (34.1)		
**Marital status**, ***n*** **(%)**					83.53	< 0.001
Married	138 (61.9)	153 (77.7)	211 (90.2)	195 (92.4)		
Non–married^b^	85 (38.1)	44 (22.3)	23 (9.8)	16 (7.6)		
**Smoking status**, ***n*** **(%)**					1.16	0.762
Smoking	48 (21.5)	45 (22.8)	58 (24.8)	44 (20.9)		
Non–smoker	175 (78.5)	152 (77.2)	176 (75.2)	167 (79.1)		
**Alcohol intake**, ***n*** **(%)**					0.11	0.991
Drinking	43 (19.2)	36 (18.3)	45 (19.2)	41 (19.4)		
Non–drinker	180 (80.7)	161 (81.7)	189 (80.8)	170 (80.6)		
**BMI**, ***n*** **(%)**					9.51	0.392
< 18.5	8 (3.6)	99 (44.4)	93 (41.7)	23 (10.3)		
18.5–23.9	10 (5.1)	85 (43.2)	80 (40.6)	22 (11.2)		
24.0–27.9	11 (4.7)	115 (49.2)	73 (31.2)	35 (15.0)		
≥28.0	6 (2.8)	104 (49.3)	76 (36.0)	25 (11.9)		
**Hypertension**, ***n*** **(%)**					5.82	0.121
Yes	157 (70.4)	144 (73.1)	147 (62.8)	143 (67.8)		
No	66 (29.6)	53 (26.9)	87 (37.2)	68 (32.2)		
**Diabetes**, ***n*** **(%)**					1.39	0.707
Yes	45 (20.2)	33 (16.8)	45 (19.2)	35 (16.6)		
No	178 (79.8)	164 (83.3)	189 (80.8)	176 (83.4)		

a Data are n (%) unless indicated otherwise; MMSE, mini–mental state examination; BMI, body mass index.

b non–married means single, divorced, or widowed.

### The relationship between social support and cognitive function

As shown in Table 3, in multiple linear regression analysis of controlling the demographic characteristics, lifestyle, and history of diseases, compared to the participants with the lowest quartile of social support score (Q1), those with the highest quartile of social support score (Q4) was positively related to MMSE score (β = 0.86, 95% *CI*: 0.19, 1.53). The β for 1-unit increment of social support was 0.05 (95% *CI*: 0.02, 0.09; *P* < 0.001) for MMSE, showing a positive relationship between social support and MMSE scores. In addition, objective support and subjective support scores were not related to the MMSE scores. The support utilization score was positively related to MMSE scores, and the β was 0.14 (95% *CI*: 0.04, 0.24) for 1-unit increment (Table 4).

**Table 3:** Relationship between the social support and score in MMSE in multiple linear regression analysis^a^.

	**Social support**				**1 unit increment**	***P* for 1 unit increment**
	**Quartile 1**	**Quartile 2**	**Quartile 3**	**Quartile 4**		
Model 1^b^	0 (ref.)	0.04 (−0.66, 0.73)	**0.78 (0.10**, **1.45)**	**1.59 (0.90**, **2.28)**	**0.10 (0.06**, **0.14)**	< 0.001
Model 2^c^	0 (ref.)	−0.20 (−0.86, 0.44)	0.27 (−0.37, 0.91)	**0.86 (0.19**, **1.53)**	**0.06 (0.02**, **0.10)**	< 0.001
Model 3^d^	0 (ref.)	−0.22 (−0.88, 0.43)	0.29 (−0.35, 0.94)	**0.86 (0.19**, **1.53)**	**0.05 (0.02**, **0.09)**	< 0.001

a Data are multivariate β (95% confidence interval); support score range of quartile 1–4 corresponds to 12–28, 29–32, 33–36, and 37–53, respectively.

b Model 1: adjusted for age and sex.

c Model 2: adjusted for model 1+ education level, marital status, smoking status, and alcohol intake.

d Model 3: adjusted for model 2+ body mass index, hypertension, and diabetes.

Bold values means *P* < 0.05.

**Table 4:** The relationship between three domains of social support and MMSE score in multiple linear regression^a^.

**Domains**	**β of 1 unit increment**	***P* for 1 unit increment**
Objective support		
Model 1^b^	**0.13 (0.03, 0.23)**	0.013
Model 2^c^	0.05 (−0.04, 0.15)	0.293
Model 3^d^	0.05 (−0.05, 0.14)	0.355
Subjective support		
Model 1^b^	**0.10 (0.04, 0.16)**	0.002
Model 2^c^	0.06 (−0.04, 0.11)	0.067
Model 3^d^	0.06 (−0.04, 0.11)	0.067
Support utilization		
Model 1^b^	**0.22 (0.12**, **0.33)**	< 0.001
Model 2^c^	**0.14 (0.04**, **0.24)**	0.005
Model 3^d^	**0.14 (0.04**, **0.24)**	0.005

a Data are multivariate β (95% confidence interval).

b Model 1: adjusted for age and sex.

c Model 2: adjusted for model 1+ education level, marital status, smoking status, and alcohol intake.

d Model 3: adjusted for model 2+ body mass index, hypertension, and diabetes.

Bold values means *P* < 0.05.

### Association of social support with cognitive impairment

As shown in Table 5, by the logistic regression model, the associations of social support with cognitive impairment were examined after adjusting the covariates. In the analysis of adjusted model 3, compared to the participants with Q1, those with Q4 have a reduced odds ratio (*OR* = 0.50, 95% *CI*: 0.32, 0.80). With the 1-unit increment of social support score, there was a 5% decreased risk (*OR* = 0.95, 95% *CI*: 0.92, 0.98; *P* = 0.003) for cognitive impairment. Moreover, the higher scores in support utilization were associated with lower risks of CI, with a 10% decreased risk (*OR* = 0.90, 95% *CI*: 0.84, 0.96) for a 1-unit increment (Table 6).

**Table 5:** Association between the social support and cognitive impairment in logistic regression.

	**Social support**				**1 unit increment**	***P* for 1 unit increment**
	**Quartile 1**	**Quartile 2**	**Quartile 3**	**Quartile 4**		
NC/CI, *n* / *n*	140/83	118/79	176/58	167/44		
Model 1^b^	1.00 (ref.)	1.19 (0.80, 1.78)	**0.62 (0.41**, **0.93)**	**0.50 (0.32**, **0.77)**	**0.95 (0.93**, **0.97)**	< 0.001
Model 2^c^	1.00 (ref.)	1.20 (0.80, 1.80)	**0.62 (0.41**, **0.96)**	**0.50 (0.32**, **0.80)**	**0.95 (0.92**, **0.98)**	0.003
Model 3^d^	1.00 (ref.)	1.21 (0.80, 1.82)	**0.62 (0.40**, **0.94)**	**0.50 (0.32**, **0.80)**	**0.95 (0.92**, **0.98)**	0.003

a Data are odds ratio (95% confidence interval); support score range of quartile 1–4 corresponds to 12–28, 29–32, 33–36, and 37–53, respectively; NC, normal cognition; CI, cognitive impairment.

b Model 1: adjusted for age and sex.

c Model 2: adjusted for model 1+ education level, marital status, smoking status, and alcohol intake.

d Model 3: adjusted for model 2+ body mass index, hypertension, and diabetes.

Bold values means *P* < 0.05.

**Table 6:** The association of three domains of social support with cognitive impairment in logistic regression^a^.

**Domains**	***OR* of 1 unit increment**	***P* for 1 unit increment**
Objective support		
Model 1^b^	0.94 (0.89, 1.00)	0.063
Model 2^c^	0.96 (0.90, 1.02)	0.158
Model 3^d^	0.96 (0.90, 1.02)	0.213
Subjective support		
Model 1^b^	**0.94 (0.91, 0.98)**	0.001
Model 2^c^	**0.94 (0.91, 0.98)**	0.003
Model 3^d^	**0.94 (0.91, 0.98)**	0.003
Support utilization		
Model 1^b^	**0.90 (0.80**, **0.96)**	0.002
Model 2^c^	**0.90 (0.84**, **0.96)**	0.002
Model 3^d^	**0.90 (0.84**, **0.96)**	0.002

a Data are odds ratio (95% confidence interval).

b Model 1: adjusted for age and sex.

c Model 2: adjusted for model 1+ education level, marital status, smoking status, and alcohol intake.

d Model 3: adjusted for model 2+ body mass index, hypertension, and diabetes.

Bold values means *P* < 0.05.

## Discussion

We examined the relationship between social support and cognition among older adults in China, and found that the level of social support, particularly in support utilization, was positively associated with cognitive function. In addition, higher levels of social support, subjective support, and support utilization were associated with reduced risks of cognitive impairment, after controlling the socio-demographic characteristics, lifestyle factors, and history of diseases.

Our results are in accordance with previous studies (12, 17–22), investigating the link between social support and cognitive function. A cohort study of 624 older adults using the structural equation model showed that social support at baseline was negatively related to cognitive function 2 years later (22). A longitudinal study, including 2,255 participants aged 55–85 over 6 years of follow-up, found that high levels of emotional and instrumental support were related to better cognitive performance (17). A cross-sectional study also showed the positive relationship between social support and cognitive function in middle-aged African Americans (12), older adults in China (20), Mexican adults aged 50 and older (18), and middle-aged and older adults in rural South Africa (19). Another study, consisting of 623 middle-aged adults with a family history of Alzheimer's disease, reported that a higher level of social support was associated with better performance on tests of speed and flexibility (21). A large-scale brain image study found that individuals with social isolation had lower gray matter volumes in several brain regions temporal, such as the temporal lobe, frontal lobe, and hippocampus, and the gray matter volumes partly explained the association of baseline social isolation with cognition (16). However, several studies indicated different results from this study. Prospective studies (13, 23) have explored the temporal associations between cognitive function and social support and showed that poor cognitive function might have a negative effect on social support, and social support in mid-life was not associated with outcomes of cognitive impairment in late life. Further interventional studies and long-term observational studies are needed to prove the causal association and temporal relationship between social support and cognitive function.

The present study also indicated the inverse relationship between cognitive impairment and both subjective support and support utilization in older adults. The items of subjective support reflected the magnitude of emotional support from friends, neighbors colleagues, and family members (38). Consistent with our results, a longitudinal study with over 7.5 years of follow-up found a significant relationship between the high level of emotional support and change in global cognitive function. The aforementioned longitudinal (17) and cross-sectional (12, 19) studies also showed similar associations between emotional support with cognitive function. The items of support utilization describe the degree of voluntarily seeking others' help and understanding and attending social activities, which may involve the willingness to communicate and collaborate with others. In addition, the objective support was not related to cognition function. These associations of the sub-domain of social support and cognitive function highlighted the importance of emotional support and support utilization, suggesting that facilitating the emotional support and support utilization of participants may be beneficial to the prevention of poor cognitive performance and cognitive impairment.

Some interpretations may increase the understanding of the relationship between social support and cognitive function. Accumulating evidence demonstrated that social support was associated with a reduced risk of depression (39, 40) and well-being (41). Several large prospective studies proved that these improvements in mental health might delay the progression of cognitive impairment and Alzheimer's disease (42). Moreover, social support also means the degree of feedback when older adults ask for needs and help. For instance, foods to eat and drugs to perform always need to be provided and prepared for older adults. The loss of these may cause accelerating cognitive decline. Importantly, as vulnerable people, older adults have more benefits from social support than younger. In addition, social support could reflect long-term care from others, which may increase the management of blood lipids and glucose (8–11) and reduce the risk of Alzheimer's disease and dementia in late life for the benefit of controlling blood glucose and lipids well.

The biological mechanisms that social support is beneficial to cognitive function are feasible. Social interaction was considered as a pathway of mental stimulation, which could contribute to cognitive reserves by activating and strengthening neurobiological activities (18, 43). Animal studies also indicated that the level of brain-derived neurotrophic factor (BDNF) playing a beneficial role in the brain was related to social support in rats (44, 45). Interestingly, another human study showed that social support from others was associated with BDNF, which alleviates the stress response (46). The evidence from human studies and animal experiments suggested that tDNF may explain the links between social support and cognitive function.

In addition, the specialized questionnaire was important to evaluate the level of social support. This study used the SSRC to assess the social support of participants. The items of SSRC consist of objective support, perceived social support, and support utilization, which comprehensively represent the level of social support. SSRC was confirmed with good reliability and α's coefficient (47, 48). Epidemiological studies among the Chinese population have widely used SSRC to examine the relationship between social support and diseases (32, 49). Over past decades, studies across the world have employed various tests to determine social support. However, there are some differences between the various versions of social support due to the distinction of culture and language among different countries and areas (13, 17, 19). Thus, future studies using approximate questionnaires will contribute to providing empirical evidence to evaluate the association of social support with diseases.

This study has several limitations to report. First, given the cross-sectional nature of this study, we could not examine the temporal association between social support and cognitive function. Second, the MMSE, as the assessment of cognitive function, was used in this study. Further epidemiological studies employing a battery of multi-domain cognitive tests and clinical diagnosis of MCI and dementia as outcomes are promising. Third, all participants in this study were recruited from Hubei province, and the results may not apply to older adults in other parts of China. Fourth, we did not test the biomarkers associated with support and cognition, such as BDNF. Further studies based on blood or brain biomarkers are needed to confirm the findings in this study. Fifth, although we have adjusted for many potential confounders in this study, we are unable to completely rule out residual confounders from unmeasured factors.

## Conclusions and implications

In this cross-sectional study, we found that social support and support utilization were positively related to cognitive function. In addition, social support, subjective support, and support utilization were negatively associated with the risk of cognitive impairment. The improvement of social support, particularly in support utilization, from health care policies, society, and family numbers may have a protective effect on late-life cognition among old adults in China.

## Data availability statement

The raw data supporting the conclusions of this article will be made available by the authors, without undue reservation.

## Ethics statement

The studies involving human participants were reviewed and approved by Medical Ethics Committee of Medical College, Wuhan University of Science and Technology. The patients/participants provided their written informed consent to participate in this study.

## Author contributions

SR and XL proposed and designed the study. SR and BL drafted this article. YG and BL completed the statistical analysis. SR supervised the data acquisition, statistical analysis, and interpretation of results. All authors critically revised the manuscript for important intellectual content.

## Funding

This work was supported by the Health Commission of Hubei Province's Scientific Research Project (Grant No. WJ2019H306) and the National Natural Science Foundation of China (No. 81941016). The funders of the study had no role in the study design, data interpretation, writing of the report, or decision of a publication.

## Conflict of interest

The authors declare that the research was conducted in the absence of any commercial or financial relationships that could be construed as a potential conflict of interest.

## Publisher's note

All claims expressed in this article are solely those of the authors and do not necessarily represent those of their affiliated organizations, or those of the publisher, the editors and the reviewers. Any product that may be evaluated in this article, or claim that may be made by its manufacturer, is not guaranteed or endorsed by the publisher.
